# Supplementary material for: The wings before the bird: an evaluation of flapping-based locomotory hypotheses in bird antecedents
Source: PeerJ. 2016 Jul 7;4:e2159. doi: 10.7717/peerj.2159 (PMC4941780; doi:10.7717/peerj.2159)
Supplement: Supplemental Information 4 — Explanation for equations used. [file peerj-04-2159-s004.docx]

Explanation of equations used during this study

Please see attached spreadsheet (called **Calculations**), which includes equations for weight supported during W.A.I.R. / leaping takeoff and for height gain through flap leaping. We have included data from the smaller specimen of *Anchiornis* to provide a guide. User inputted values are highlighted in **bold** to differentiate them from calculated values. As alterations to any of these values will change the resulting body mass support and leaping height we intentionally choose values at the higher end on the spectrum either from extant avians or from previous estimates of theropod morphology to ensure we were gaining maximum outputs from our analyses. Flap running values were based on the equation in Burgers and Chiappe (1999) and modified as stated in the methods section.

Body mass in Non-avian theropods was based on femur length following equation from Christiansen and Farina (2004).

log_10_ mass=3**.**222 (+/- 0**.**181)log_10_ FL - 6.288 (+/- 0**.**500)

Femoral length was chosen as it has been previously shown to be a stable proxy (Dececchi and Larsson 2013). As this relationship is broken in early avians this equation was not used for taxa at the level of Archaeopteryx and up.

For avian theropods there is less consensus on a good mass estimation model, so we chose two recent studies to give “heavy” and “light” values to bracket the performance scores in our analysis. These estimates are both based on humeral length due to its availability across taxa studied and its strong fidelity as a body mass proxy.

From Liu et al. 2012

log_10_ mass=1.73328 log_10_ FL – 0.76280

From Field et al. 2013

Ln mass=2.07(Ln HL)-2.77

**Wing beats**

Flapping frequency (*f*) values were estimated from extant avians performance during take off to gain values that most closely approximate conditions expected during W.A.I.R. Equations for ALL and GF were taken from the maximum wing beat equations in Jackson 2009. This was chosen to give the highest possible value and thus is likely gross overestimation. Using mean values reduces frequency estimates and thus the body weight support values, making it less likely that non-paravians could perform W.A.I.R. and therefore does not significantly alter our conclusion.

All

log_10_ frequency =-0.38log_10_ FL + 0.79

GF

log_10_ frequency =-0.38log_10_ FL + 0.75

MOD was produced by combining the wingbeat data from Jackson 2009 and Askew et al. 2001 to include Galliformes to make it more comparable to W.A.I.R. derived from Chuckars and other members of this clade.

**Body weight support calculations**

Flap Amplitude is calculated by multiplying flap angle (Θ) in radians by wing length in meters (b)

Amp=Θ* b

This produces an estimate of arc length as opposed to chord length, which results in a overestimation of amplitude.

Strouhal number

St= *f*Amp/U

Where U is the body velocity, taken here for W.A.I.R. as that seen in adult Chuckars (Tobalske and Dial 2007) of 1.5m/s. For calculations of leaping take off we used body velocities that are seen during leaping takeoffs in extant avians with values of 3.8 m/s as seen in quails (Earls 2000) and 4.1m/s from both Turkey and Harris Hawk (data from Tobalske and Dial 2000, Askew et al. 2001, Heers et al. 2014). We also included an upper estimate of 5.1 m/s that has been calculated for high powered leapers such as Galagos (Gunther et al. 1991).

To calculate the percentage of body weight supported by any flapping based motion we used modified equations from Burgers and Chiappe (1999)

Bw=0.5Cl**p* *(*f*Amp +U)^2^ S/9.8M

Where Cl is the coefficient of lift, and here taken for W.A.I.R. to be 1 based on the value seen in immature Chuckars (Heers et al. 2011) and for flapping take off at 1.5 based on that seen in adult Chuckars at high level W.A.I.R. (Heers et al. 2011) and slightly below that seen in avian take off (Underwood 2009). *p* is the density of air, here set at 1.23. *f* is flapping frequency, Amp is flap amplitude and U is the body velocity. S is wing area in m^2^ and M is body mass in kg.

**Leap height**

Hip height (h_hip_ ) was based on the additive linear dimensions of the femur+ tibia+ metatarsus. To account for crouching we multiplies this value by 0.8 which mimics the level seen in modern ostriches (Birn-Jeffery et al. 2014), a highly cursorial bird whose upright stance was used as a proxy for non-avian theropods.

For height gained during leaping we needed to generate first the parameters of a leaping takeoff based on the work of Witton and Habib (2010).

Unload distance D_un_=0.75*h_hip_

Flap time (t_fl_)=1/3.98*M^-0.27^ where M equals body mass

Launch time (t_launch_)= t_fl_ LS

Where LS is the launch scalar require to generate a preloading value to 2.4. This preloading value is taken from Bieweiner 2003 based on extant taxa to represent a moderately capable leaping organism.

Preloading itself is calculated as Power _required_ / Power _output_

Where Power _required_=MV_bal_A+g

Where A is acceleration during the leap calculated as

A=V_bal/_ t_launch_

Ballistic velocity (V_bal_= D_un /_t_launch)_

Height _unaided_ =V_bal_ ^2^ sin(Φ_launch_)^2^/2g

Where g=9.8m/s^2^ and Φ_launch_ is the launch angle.

Power _output_= (150 (1 –Muscle _anaerobic_))+ (400 Muscle _anaerobic_) Muscle _hind_

Muscle _anaerobic_ is denotes the proportion of the muscle that is anaerobically “fast twitch” muscles that are responsible for explosive movement. For the hindlimb this proportion was set at 20% of total muscle volume, while for the forelimb it is set at 50%. Both these values represent the higher end of values seen in extant avians (Butler 1991).

Muscle _hind_ is the proportion of body mass devoted to hindlimb muscles including the M. caudofemoralis a tail muscle that acts as a major femoral retractor in theropods. We set this value at a constant 30% of total mass for hindlimbs and 10% fore forelimbs (Muscle _fore_). Both of these are likely an overestimation and were selected to provide an upper boundary value to ensure we did not underestimate the potential leaping ability of these taxa.

Height _flap_ =V_combined_ ^2^ sin(Φ_launch_)^2^/2g

Where V_combined_ = V_bal_ + V_downstroke_ (*f*/jump duration)

**References:**

Askew, G. N., R. L. Marsh, and C. P. Ellington. 2001. The mechanical power output of the flight muscles of blue-breasted quail (Coturnix chinensis) during take-off. J. Exp. Biol. 204:3601–3619.

Biewener, A. A. 2003. Animal locomotion. Oxford University Press.

Birn-Jeffery, A.V., Hubicki, C.M. Blum, Y., Reniewski, D. Hurst, J.W., and M.A. Daley. 2014. Don’t break a leg: running birds from quail to ostrich prioritise leg safety and economy on uneven terrain. J. Exp. Biol. 217:3786–3796.

Burgers, P., and L. M. Chiappe. 1999. The wing of Archaeopteryx as a primary thrust generator. Nature 399:60–62.

Butler, P.J. 1991. Exercise in birds. J. Exp. Biol. 160:233–262.

Christiansen, P., and R. A. Farina. 2004. Mass Prediction in Theropod Dinosaurs. Hist. Biol. 16:85–92.

Dececchi, T. A., and H. C. E. Larsson. 2013. Body and limb size dissociation at the origin of birds: uncoupling allometric constraints across a macroevolutionary transition. Evolution 67:2741–2752.

Earls, K. D. 2000. Kinematics and mechanics of ground take-off in the starling Sturnis vulgaris and the quail Coturnix coturnix. J. Exp. Biol. 203:725–739.

Field, D. J., C. Lynner, C. Brown, and S. A. F. Darroch. 2013. Skeletal correlates for body mass estimation in modern and fossil flying birds. PLoS One 8:e82000.

Günther, M. M., H. Ishida, H. Kumakura, and Y. Nakano. 1991. The jump as a fast mode of locomotion in arboreal and terrestrial biotopes. Z. Morphol. Anthropol. 78:341–372.

Heers, A. M., B. W. Tobalske, and K. P. Dial. 2011. Ontogeny of lift and drag production in ground birds. J. Exp. Biol. 214:717–725.

Jackson, B. E. 2009. The allometry of bird flight performance. etd.lib.umt.edu.

Liu, D., Z. H. Zhou, and Y. G. Zhang. 2012. Mass estimate and evolutionary trend in Chinese Mesozoic fossil birds. Vertebr. Palasiat 50:39–52.

Witton, M. P., and M. B. Habib. 2010. On the Size and Flight Diversity of Giant Pterosaurs, the Use of Birds as Pterosaur Analogues and Comments on Pterosaur Flightlessness. PLoS One 5:e13982.

Tobalske, B. W., and K. P. Dial. 2007. Aerodynamics of wing-assisted incline running in birds. J. Exp. Biol. 210:1742–1751.

Usherwood, J. R. 2009. The aerodynamic forces and pressure distribution of a revolving pigeon wing. Exp. Fluids 46:991–1003.
